# Global Development of Anticancer Therapies for Rare Cancers, Pediatric Cancers, and Molecular Subtypes of Common Cancers

**DOI:** 10.1200/JGO.18.00092

**Published:** 2018-12-06

**Authors:** H. Kim Lyerly, Jun Ren, Renzo Canetta, Gi Hyun Kim, Sumimasa Nagai, Tomohiro Yamaguchi, Ken Hatogai, Hiroshi Katayama, Silvy Da Rocha Dias, Daniel McManus, Kathy Soltys, Zhimin Yang, Olufumilayo Olopade, Nancy Goodman, Greg Reaman, Thomas Gross

**Affiliations:** **H. Kim Lyerly**, Duke University, Durham, NC; **Jun Ren**, Capital University Medical Center; **Zhimin Yang**, China Food and Drug Administration, Beijing, People’s Republic of China; **Gi Hyun Kim**, Korea Ministry of Food and Drug Safety, Seoul, South Korea; **Sumimasa Nagai**, University of Tokyo; **Sumimasa Nagai**, **Tomohiro Yamaguchi**, and **Ken Hatogai**, Pharmaceuticals and Medical Devices Agency; **Hiroshi Katayama**, National Cancer Center Hospital, Tokyo, Japan; **Silvy Da Rocha Dias**, European Medicines Agency, London, United Kingdom; **Daniel McManus** and **Kathy Soltys**, Health Canada, Ottawa, Ontario, Canada; **Olufumilayo Olopade**, University of Chicago, Chicago, IL; **Nancy Goodman**, Kids V Cancer, Washington, DC; **Greg Reaman**, US Food and Drug Administration; **Thomas Gross**, US National Cancer Institute, Bethesda, MD, and **Renzo Canetta**, Independent Consultant.

## Abstract

Advances in genetic sequencing and other diagnostic technologies have enabled the use of precision medicine in clinical cancer care, as well as the development of novel therapies that are targeted to specific molecular drivers of cancer. Developing these new agents and making them accessible to patients requires global clinical studies and regulatory review and approval by different national regulatory agencies. Whereas these global trials present challenges for drug developers who conduct them and regulatory agencies who oversee them, they also raise practical issues about patients with low-frequency cancers who need these therapies. A lack of uniform standards in both regulatory approval for marketing and reimbursement for approved agents across countries may make the newly developed agent either unavailable or inaccessible to patients in certain countries or regions, even if patients from those countries or regions participated in the clinical research that established the safety and efficacy of the agent. In an effort to further understand and address this need, we convened an international workshop in 2017 in North Bethesda, MD. After presentations of the individual regulatory pathways for marketing approval and reimbursement for individual nations, participants discussed expedited pathways and specific challenges for uncommon cancers. As a matter of justice, agents being developed for rare cancers, pediatric cancers, or uncommon molecular subsets of common cancers need a pragmatic, science-based regulatory policy framework to clearly specify the type and quantity of evidence needed to demonstrate efficacy from these trials and evidence to support accessibility.

## INTRODUCTION

Before patients can use new anticancer drugs in most developed economies, the appropriate regulatory authority, typically the US Food and Drug Administration (FDA) in the United States, reviews the clinical trial results to confirm that the benefits outweigh the harms for the indication.^1^ For oncology products in the United States, a recently created FDA Oncology Center of Excellence coordinates these reviews.^2^ Recent advances in genetic sequencing and other diagnostic technologies has enabled the use of precision medicine in clinical cancer care, as well as the development of novel therapies targeted to specific molecular drivers of cancer.^3,4^ Researchers are now characterizing different forms of cancer on the basis of shared genomic changes, in essence molecularly subdividing cancer types. The result of this molecular reclassification is that more cancers—even some forms of common cancers, such as lung cancer—can be labeled as rare. Because identifying patients with rare cancers is more difficult—sometimes requiring screening hundreds of patients to enroll eligible participants—it is often mandatory to involve centers throughout world to be able to complete clinical trials. We sought to understand the regulatory pathways for the development of modern anticancer agents in this new era.

**Fig 1:**
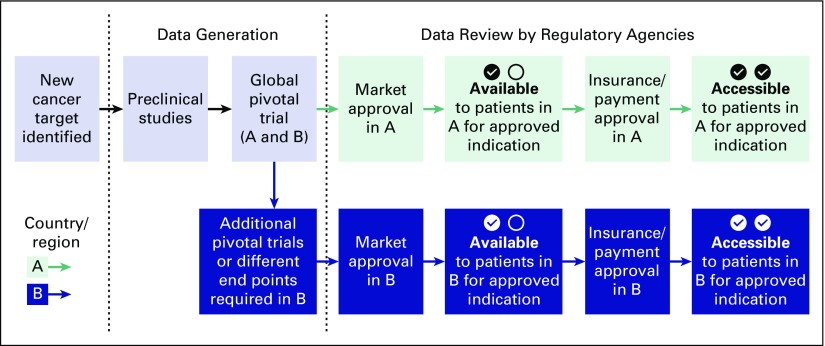
The regulatory pathway to marketing approval and reimbursement for oncology products. Once a new cancer target is identified, preclinical studies are conducted to develop a new agent. Once sufficient data are generated to justify human studies, clinical trials are conducted to determine the safety and efficacy of the new agent, usually sponsored and financed by company or industry. Often, phase III clinical trials are conducted to demonstrate the safety and efficacy for a specific cancer and indication, which are referred to as pivotal trials. Once completed, the data from these trials are reviewed by the appropriate regulatory authorities (A: US Food and Drug Administration [FDA]) and a decision is made about the safety and efficacy of the new agent. If FDA marketing approval is granted for the specific indication, the US agencies and insurers that are responsible for providing reimbursement begin to pay for the new agent when administered for the approved indication. If the sponsoring company would like to provide this product to patients in other parts of the world, such as Europe or Japan, the original data and often additional data from other phase III clinical trials for the same patients and indication are submitted to the appropriate national or regional regulatory authority for marketing approval (B: European Medicines Agency or Pharmaceuticals and Medical Devices Agency, respectively). Marketing approval is then followed by review and a decision about providing reimbursement to patients by the national health service, health authorities, and/or insurers of the respective countries.

A template of a common regulatory pathway for oncology products is shown in Figure 1, with the United States described as the first market granting approval, followed by additional nations and regions. In different cases, other nations or regions may grant the first approval of an oncology product, followed by the United States and other nations.^5^

This system results in a sequence of approvals for marketing and reimbursement that can result in a marked difference in drug accessibility throughout the world. Whereas sponsors and regulatory agencies have leveraged knowledge about molecular drivers of cancer to develop innovative strategies to codevelop new therapeutic agents and the appropriate biomarkers to select patients who are most likely to respond,^6^ efficient strategies to evaluate the effects of an investigational agent and the subsequent clinical outcome are challenging when the specific molecular alterations occur infrequently.^7^ This is especially important today, as 90% of all cancer drugs that have been developed in the past 5 years have been for the treatment of rare cancers, which allows them to have orphan drug status in the United States. Expansion of the number and definition of rare diseases and the needs for children with cancer highlights the need to understand obstacles to therapeutic development where the number of patients who can enroll in studies is limited.

**Fig 2:**
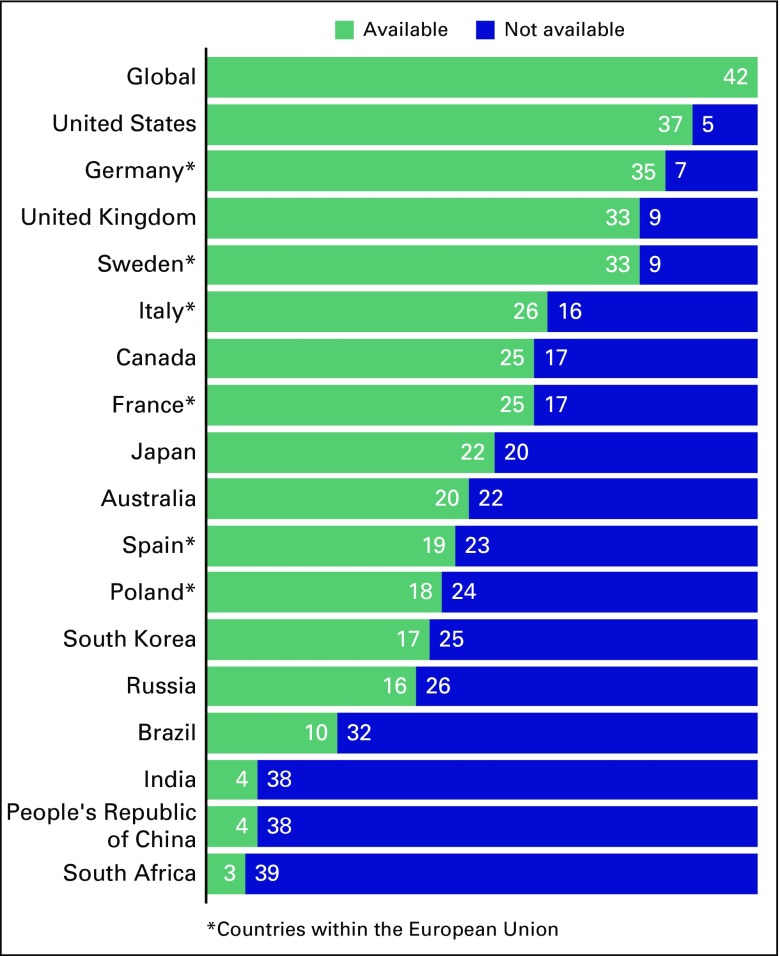
Availability in 2016 of new oncology drugs launched initially during 2011 to 2015. Adapted from IQVIA.^9^

Whereas challenges for global drug development exist for both drug developers and regulatory agencies, an additional barrier exists for those who develop therapies for patients with low-frequency cancers. A lack of uniform standards in both regulatory approval for marketing and reimbursement for approved agents across countries may make the newly developed agent either unavailable, inaccessible, or delayed for long periods of time to patients in certain countries or regions, even if patients from those countries or regions participated in the clinical research that proved the safety and efficacy of the agent.^8^ Although expert reviewers exist within the national regulatory authorities around the world, the specific national or regional regulatory requirements for marketing and reimbursement approval are not uniformly harmonized. This makes it nearly impossible for clinical data from pivotal trials of patients demonstrating the efficacy and safety of a new agent to result in approval by all regulatory authorities for marketing or reimbursement in all countries or regions. In an assessment of 42 oncology drugs that were launched globally from 2011 to 2015, availability ranged from 37 of 42 being available in the United States 19 to 35 of 42 being available in countries in the European Union—represented by data from Germany, Sweden, Italy, France, Spain, and Poland—to 22 of 42 being available in Japan and 10 or fewer being available in such countries as Brazil, India, People’s Republic of China, and South Africa, as shown in Figure 2.

The global variability of availability and accessibility of an effective new agent is usually resolved in more common diseases by sophisticated development plans that include the initiation of parallel studies to demonstrate safety and efficacy with different study designs or end points in specific populations, or studies conducted in specific nations designed to meet specific regulatory requirements. Unfortunately, for less common diseases, this may not be practical or possible, as there may not be sufficient numbers of patients to conduct additional studies in a realistic period. The consequences of this situation are enormous for patients with uncommon cancers, as a positive study outcome that results in the availability and accessibility of the new agents in only a single market may make similar studies, designed to meet the regulatory requirements for another country or region, impossible to conduct. Opportunities to use real-world data and/or sharing data from clinical studies may help address this issue, but understanding the process and considering opportunities to harmonize criteria may also be useful.

As a matter of justice, communities of participants in research are entitled to some benefit. Consequently, in developing agents for rare cancers, pediatric cancers, or uncommon molecular subsets of common cancers, there is a need for a pragmatic, science-based regulatory policy framework to clearly specify the type and quantity of evidence needed to demonstrate efficacy from these trials. Because of this need, we convened a workshop of international participants in 2017 to begin a dialogue to understand and address this issue.

## METHODS

Using a successful model of educational interaction between academia, government, and industry in oncology regulatory science,^10^ we invited representatives of relevant regulatory agencies or an experienced drug development professional and/or clinical investigator working in that country or region to a 1-day workshop in North Bethesda, MD, that focused on drug development for rare cancers, pediatric cancers, or uncommon molecular subsets of common cancers (Table 1).

**Table 1:**
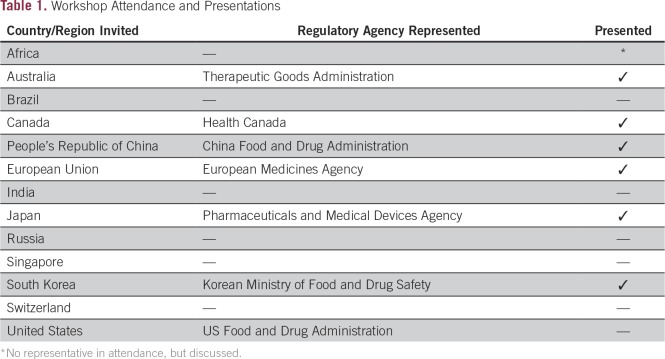
Workshop Attendance and Presentations

Presenters were asked to provide a description of the regulatory process for marketing approval and reimbursement for oncology products in their country or region. Presenters also addressed how oncology products were developed for rare, pediatric or molecular subsets of common cancers. Invited presenters and the countries and regions represented are listed in Table 1.

Speakers presented the process of cancer drug development and reimbursement in their country or region. Specific highlights included strategies to improve the process to address rare cancers, pediatric cancer, and uncommon molecular subsets. A moderated discussion after each presentation focused on the aspects of the individual regions that could be opportunities to enhance success in the development of effective new agents.

## RESULTS

### Regulatory Oversight for Marketing Approval and Reimbursement in Various Countries and Regions

As shown in Tables 1 and 2, presentations from the United States, European Union, Japan, People’s Republic of China, South Korea, Canada, and Australia were made. A specific session was devoted to cancer drug development and research in Africa. Each of the presentations provided a general description of the process for regulatory approval after review of the clinical data generated to support new drug applications. Although it is widely appreciated that the standard drug development timeline may span years, it was clear that the overall process of review of the data for each agency in the original regulatory review accounted for a relatively small portion of this time. Review and negotiation of pricing would add additional time. Nonetheless, participants described various strategies to improve the oncology drug development process, including expedited programs and various approval pathways. Finally, participants discussed the role of regulatory review and separate review and decisions regarding reimbursement decisions when appropriate.^11-13^

**Table 2:**
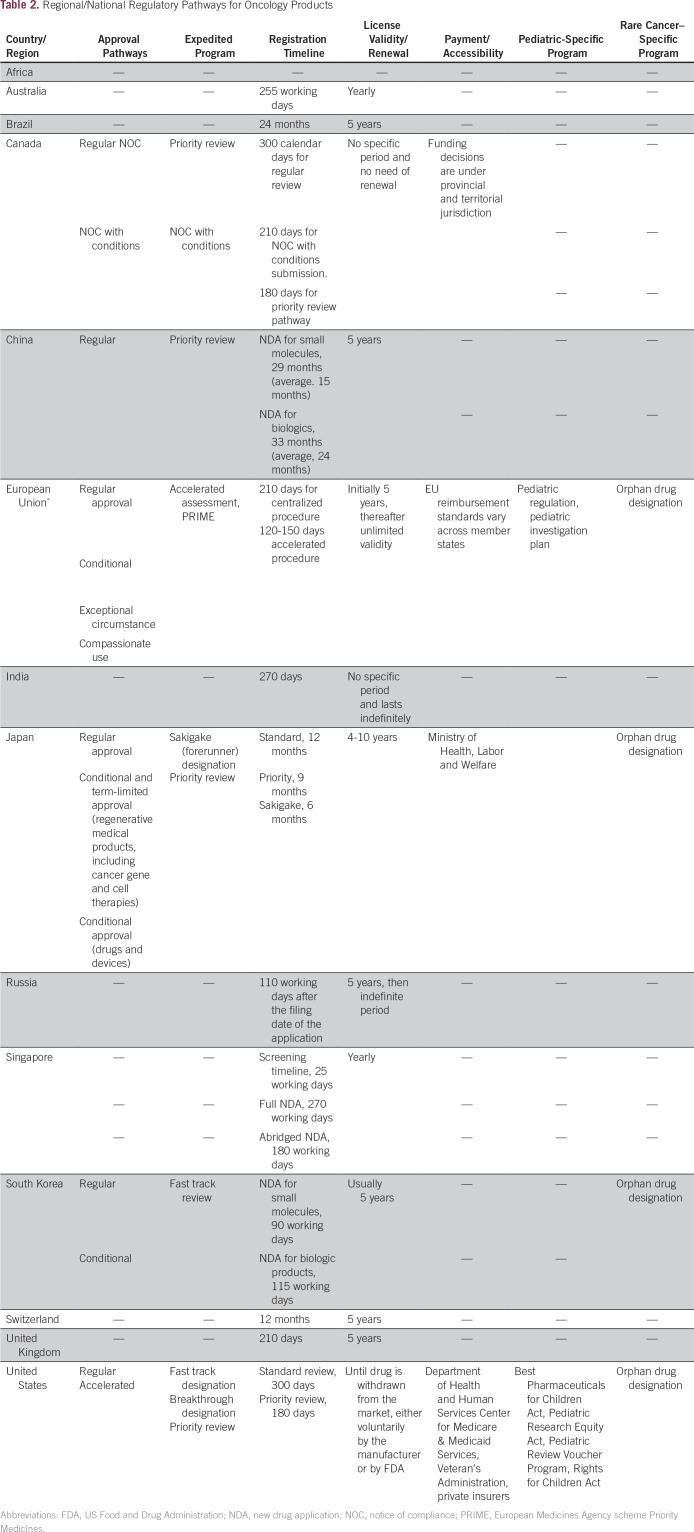
Regional/National Regulatory Pathways for Oncology Products

Despite the fact that individual review processes were relatively short, challenges to approval emerged as a result of their variability. The regulatory requirements, structure, and processes of various regulatory authorities differ across countries, which makes it challenging for pharmaceutical companies to develop drugs for simultaneous submission to all regulatory authorities. Regulatory requirements for emerging oncology products are also changing in some agencies, and it is important to understand the similarities and differences between regulatory requirements to accelerate the marketing of new anticancer products. To share information and understand similarities and differences between regulatory frameworks, monthly international regulatory teleconferences among some regulatory agencies, including the FDA, European Medicines Agency (EMA), Health Canada, and Pharmaceuticals and Medical Devices Agency, have been ongoing.^14^

### Challenges to Rare Cancers, Pediatric Cancers, and Molecular Subsets of Common Cancers

Whereas anticancer drug development increasingly requires global coordination, additional challenges are faced when a drug for a rare cancer is being developed.^15^ For the US National Institutes of Health and the FDA, a rare disease is defined as one with a prevalence of fewer than 200,000 affected individuals per year, or approximately one in 1,500. In addition, all cancers in children are defined as rare, as the United States has an incidence rate of 15,000 cases of pediatric cancer per year. This definition of rare can be different in different countries. For example, in the European Union and Japan, a rare cancer is defined as an annual incidence of fewer than six cases per 100,000.^16^ In contrast, there is no definition of rare disease in the People’s Republic of China as a result of insufficient epidemiologic data. Rare cancers, which affect far fewer patients, often require global cooperation to conduct a single, large, randomized clinical trial. Once complete, it might be unfeasible to repeat a study simply to meet the regulatory requirements in different geographical zones. Furthermore, results of the trial might be so compelling to physicians that they may feel it is inappropriate to ask patients to participate in a clinical trial when the evidence to support the use of the drug is clear, and it may not be possible to design a trial that could be ethically justified given the existing evidence. The United States, European Union, and Japan all have orphan drug designation programs that attempt to address challenges to developing therapeutics for patients who suffer from these disorders.

**Fig 3:**
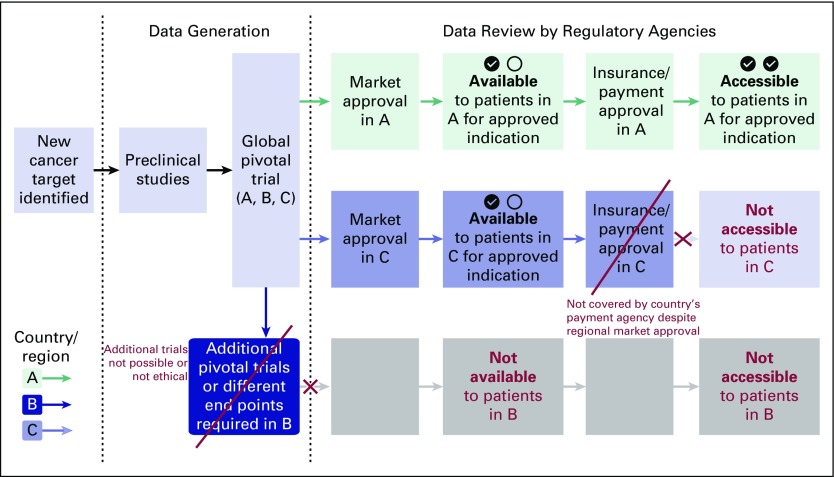
The regulatory pathway to marketing approval and reimbursement for oncology products for rare cancers and pediatric cancers. As with therapeutic agents in more common cancers, phase III clinical trials are conducted to demonstrate the safety and efficacy for a specific cancer and indication, often enrolling patients from all over the world. These data are reviewed by the appropriate regulatory authority (A: US Food and Drug Administration acts as the first reviewing regulatory agency). If there are insufficient numbers of patients to conduct additional studies, the additional data required by other agencies (B) for marketing approval may not be available, making the agent not available or accessible. In addition, even if sufficient data are available for marketing approval by C, the data necessary for coverage decision making may be available, making the agent available, but not accessible (affordable), for most patients.

The growing availability of next-generation sequencing and other molecular tools with which to define cancers has led to significant increases in the identification of putative therapeutic targets and novel moieties designed to affect these targets. Although establishing the relationship between the efficacy of an investigational agent and the specific therapeutic target in specific cancers has become an established development process, the challenges of this approach are compounded when some molecular alterations occur infrequently. This makes evaluation in clinical trials challenging because of the need to screen many patients to find eligible patients to enroll in the studies and requires significantly more time and resources. In addition, even a positive study that demonstrates the safety and efficacy of a new agent does not lead to access to the agent to all patients, as illustrated in Figure 3.

Whereas the orphan drug designation was intended to provide incentives for developing therapies for uncommon disorders, the current situation creates challenges for patients with uncommon cancers, as shown in Figure 3. If FDA marketing approval is granted for the specific indication, the US agency responsible for providing reimbursement begins to pay for the new agent administered for the approved indication. Although the sponsoring company would like to provide this product to patients in other parts of the world, the patient population available to conduct additional pivotal trials may not be available. In this case, data generated from the global clinical trials will need to be submitted to the appropriate national or regional regulatory authority for marketing approval. A significant obstacle is that the clinical trial design and clinical trial end points that are acceptable to the FDA may not be acceptable to the other regulatory authorities. Hence, the new agent may not be available or accessible to patients in these countries or regions, even though patients from these countries or regions were enrolled in the clinical trials.

## DISCUSSION

Patients are the most precious element of the drug development process. Without their voluntary participation, it would not be possible to determine the efficacy and safety of any new drug. This prominent role has justified the establishment of essential guidelines, designed to protect the rights of individuals during this process. Whereas the elements that protect individuals are a starting point, the long pathway from clinical research to regulatory approval and drug accessibility requires a broader view that encompasses the benefits and risks to the patient population across the entire process. Although the current global regulatory frameworks for cancer drug development are being evaluated to improve and optimize the development of molecularly targeted and personalized therapies, an opportunity exists to address the challenges in delivering effective therapies to patients, including children, with less common cancers.

Whereas the risks and benefits to individual patients who participate in a clinical trial have been examined, it is clear that generating clinical data is the primary goal of the activity. Consequently, the benefit to future patients is often cited as the primary imperative for participation—that is, a common belief of participants is that “this trial may not help me, but it may provide knowledge to help my children and/or others.” Hence, reliable results from high-quality clinical trials which specifically conclude that an investigational agent has been proven effective and safe are expected to benefit the populations of patients represented by the participants.^17^ In the current situation, in which heterogeneity in regulatory and reimbursement requirements disrupts this expectation, participation does not necessarily result in the new agent being readily accessible to populations that participated in the trial. It is the responsibility of the global research community to recognize this conundrum and to address this issue to deliver the promise of benefit to those participating in clinical research.

Why is now the time to address this issue? At present, developing new anticancer agents encompasses a range of activities, from hypotheses and laboratory observations, preclinical development in animal models, clinical testing in patients, regulatory approval and commercial availability, to accessibility and reimbursement for routine clinical use. This spectrum of activities requires significant time and resources, routinely estimated to be 10 years or longer, and costing more than $1 billion (USD) for the development of any one drug.^18^ There was previously a perception that the process required for the development of a promising drug was lengthy, in large part, because of complex and time-consuming regulatory oversight and review. As such, the inability of the regulatory agencies to function efficiently was cited as a barrier to developing new agents for patient care; however, presentations from various agencies confirmed that the review process itself was not a major component of the timeline to develop new oncology agents. Analysis of regulatory review time for anticancer drug studies by the FDA or EMA demonstrated that both were fairly efficient in their review of data, usually accomplishing these tasks in a few months, and were not the rate-limiting factor in the development of oncology products.^19^ Recent studies have demonstrated that physicians often have a limited understanding of the regulatory agencies and the pathways to drug approval^20^; therefore, making the regulatory review process more transparent and understandable would create an opportunity for the drug development industry to focus on best practices for developing evidence, including an understanding of common pitfalls to avoid.

In contrast to the regulatory review process, the design and conduct of clinical trials may take years to complete, as investigators must identify and enroll patients, treat them with the new agent, and observe their outcomes to generate clinical data. Improved efficiency in the design and conduct of studies would be beneficial, as the generation of reliable data would provide answers about the utility of these drugs in less time and with fewer patients. In fact, the FDA routinely provides industry guidance to support best practices for developing evidence for the safety and effectiveness of new agents. Nonetheless, enrollment of patients in clinical trials remains the major barrier to generating knowledge about the efficacy and safety of new agents.

To minimize the numbers of patients needed, sponsors have begun to use biomarkers, often enabled by genetic analysis, to identify patients who are most likely to respond to a new agent and possibly to predict the beneficial response to a new agent. The potential for higher response rates in biomarker-selected patients was anticipated to enable smaller clinical trials to demonstrate the benefit of a new agent. Indeed, the actual numbers of treated patients may be smaller, but the number of patients who are screened for the presence of the biomarker may be large, as some biomarkers are present in only a small fraction of patients. Consequently, many patients must be screened to identify even a modest-sized cohort of patients to be treated in the biomarker-directed trial. Currently, the near Herculean task of completing a clinical trial to determine the effectiveness of an anticancer agent entails usually enrolling or screening hundreds or thousands of patients, which requires opening clinical trial sites at a variety of clinics and hospitals. As a result of the need to enroll many patients as efficiently and quickly as possible, clinical trials typically include both US and international patients who are enrolled from clinics and hospitals around the world. In fact, for many new agents, many, if not most, of the patients enrolled in clinical trials are from non-US sites.

Unfortunately, the marketing approval process may not always be harmonized across all of these markets, which leads to the availability of a new drug in some markets but not others. Furthermore, a development plan and clinical trial design that might be appropriate for the FDA might not be appropriate for the other regulatory agencies around the world. In this case, a global study designed with an end point of demonstrating the safety and effectiveness of a new agent to the FDA to enable commercial entry into the US market may not be a study design deemed appropriate by the other agencies to enable commercial entry in additional markets. Consequently, whereas the drug was approved by the FDA, an additional clinical study, designed to meet the requirements of another agency, might need to be conducted, and data from this additional trial would be used to support the commercial entry of the new agent into the additional market at a later date.

Although this delay is unfortunate and may affect the availability of a new agent in additional markets, for many common cancers in which thousands of patients are available for enrollment or screening in many studies, parallel studies can be done to minimize the time in which effective agents are available in one market but not another. Hypothetically, additional large studies might be conducted, one designed for the US market and others that are designed for other markets, such as the European Union. It is more likely that the other agencies, such as the EMA, would have requirements for additional end points, the enrollment of specific patient populations, or specific analyses that would be requested and added to the study design. Nonetheless, a recent report demonstrated wide variation in the availability of effective new agents around the world. In fact, from 2010 to 2014, only a small fraction of new cancer drugs was available to all global markets. Although most agents were available in the United States and the European Union, there was a significant number of drugs not available in less developed countries.

An additional potential barrier has been raised by some advocacy groups as a result of the experiences in developing a single global clinical development strategy for pediatric cancers. A trial that is designed to meet the regulatory requirements of each country might be burdened by the restricted views of the most risk-averse country. This might impose an unnecessary burden on the development process, for example, by requiring additional safety studies, such as juvenile clinical toxicity studies. Maintaining a view that every country’s safety concerns must be addressed may prove to be cumbersome, with additional time and expense needed, which would discourage pediatric cancer drug development. Alternatives, such as agreements that countries’ regulatory agencies accept each other’s decisions for pediatric or rare disease drugs—for example, the United States and Canada could honor each other’s decisions, the United States and EMA, etc—have been proposed by some advocacy groups that seek to reduce barriers.

Finally, in addition to availability in the market, the cost of many new anticancer agents raises the question of accessibility, usually defined as being reimbursed sufficiently by government or private insurance such that a physician can routinely prescribe the use of these agents to treat their patients and ensure their health care. By law, the FDA does not consider cost in the assessment of drugs, and the Centers for Medicare & Medicaid Services typically approves the purchase of FDA-approved drugs without price negotiations with the commercial manufacturer. Other providers may follow suit. Therefore, commercial entry into the US market is a significant step toward ensuring that patients can be treated with a new drug. In contrast, in many, if not all, non-US markets, the formal process of market approval and reimbursement approval are conducted by separate agencies, and the cost of the drug can affect accessibility. In addition, these policies can affect the conduct of clinical trials, as studies may randomly assign new agents to comparator arms that should include the standard available treatment. If the standard available treatments in the comparator arm are different in different countries, the findings from the study may be difficult to interpret.

With the current rapid expansion of knowledge of cancer biology and the almost unlimited ability to develop new molecules to inhibit critical pathways in cancer, there are increasing numbers of potential anticancer agents that could be developed. Some have estimated that as many as 1,000 new molecular entities—original drugs, not copies of existing drugs—are currently being developed to fight cancer. Potential solutions to accelerate this process include alternatives to clinical trial data, such as prospective registries, or real-world data to support evidence for the efficacy and safety of new drugs and to expand their use to new indications. Other trial designs, such as umbrella and basket trials, may also accelerate our validation of the effectiveness of new agents. What has become increasingly clear is that the process to evaluate the usefulness of these drugs must be more effective and must have a global perspective if all societies are to take advantage of the current insights into the cancer process and how to prevent and treat it.
